# Supplementary material for: Chromothripsis during telomere crisis is independent of NHEJ, and consistent with a replicative origin
Source: Genome Res. 2019 May;29(5):737–49. doi: 10.1101/gr.240705.118 (PMC6499312; doi:10.1101/gr.240705.118)
Supplement: Supplemental Material [file supp_gr.240705.118_Supplemental_file_1.zip › contigs/annotated_contigs/DB51/contig.2.DB51_length_534_mean_cov_2.50374531835.docx]

**DB51_length_534_mean_cov_2.50374531835**

```
TTCTGCTATTTAATTTTAATATCTGAAAGAATTATTTCCCTGGGTGCACTTCCCCAGGCCCAGATTGCTGTTGTTCAGAGCCAAAG|AA
 >chr20:19502337-19502426 + E=7e-39 p=1e-02                                            >
T|CTGAAGGCTGTCTGGTCCCAAGCATTTGGAATAAGGGATACTCAACCTGTATATGCTTTTGCGAACTAAGTTTAGCATTTAAATGTA
chr20:7971685-7972069 - E=9e-209
TTTAAAGCATTTTTATTGGATTTGTTGAATCTCTGCTGCTCACTAATAATATAGGATGTAACTTCCCTTTCCTAAATGTTACTGTCGTT

TTGAGAGGATGTAGGCATATGGCAGATTTTAGCTAAAATAAGATGTGCTACATCCTTGGGAGGACAATCTGTGTGATGGATGCTACCTT

CCCTTTTTATTAGAATGACGTGACAGAAAACTTAAGGCTTTATTTAAAATATCTGATGATGAGAATTAAAAGCCATGAACATTTAGACT

AGAGAAGAACAAAGGGTATTTTAAAA|TATAAAAACATACTTATGTGATATAAATAGCCTGCTTATTATATATGTTTTATTATTTTAAA

ATA
```
